# Association between Use of Hydrochlorothiazide and Nonmelanoma Skin Cancer: Common Data Model Cohort Study in Asian Population

**DOI:** 10.3390/jcm9092910

**Published:** 2020-09-09

**Authors:** Seung Min Lee, Kwangsoo Kim, Jihoon Yoon, Sue K. Park, Sungji Moon, Sang Eun Lee, JiSeon Oh, Sooyoung Yoo, Kwang-Il Kim, Hyung-Jin Yoon, Hae-Young Lee

**Affiliations:** 1 Department of Biomedical Engineering, Seoul National University College of Medicine, Seoul 03080, Korea; min8607@outlook.com; 2 Transdisciplinary Department of Medicine & Advanced Technology, Seoul National University College of Medicine, Seoul 03080, Korea; kksoo716@gmail.com; 3 Washington University School of Medicine in St. Louis, St. Louis, MO 63110-1010, USA; jihoon.yoon1115@gmail.com; 4 Department of Preventive Medicine, Seoul National University College of Medicine, Seoul 03080, Korea; suepark@snu.ac.kr (S.K.P.); kajaman3@snu.ac.kr (S.M.); 5 Cancer Research Institute, Seoul National University, Seoul 03080, Korea; 6 Convergence Graduate Program in Innovative Medical Science, Seoul National University College of Medicine, Seoul 03080, Korea; 7 Interdisciplinary Program in Cancer Biology Major, Seoul National University College of Medicine, Seoul 03080, Korea; 8 Division of Cardiology, Department of Internal Medicine, Asan Medical Center, University of Ulsan College of Medicine, Seoul 05505, Korea; sangeunlee.md@gmail.com; 9 Department of Biomedical Informatics Health Innovation Big Data Center, Asan Medical Center, Seoul 05505, Korea; doogieoh@gmail.com; 10 Healthcare ICT Research Center, Office of eHealth Research and Businesses, Seoul National University Bundang Hospital, Seongnam 13620, Korea; yoosoo0@snubh.org; 11 Department of Internal Medicine, Seoul National University Bundang Hospital, Seongnam 13620, Korea; kikim907@snubh.org; 12 Department of Internal Medicine, Seoul National University College of Medicine, Seoul 03080, Korea; 13 Department of Internal Medicine, Seoul National University Hospital, Seoul 03080, Korea

**Keywords:** hydrochlorothiazide, skin cancer, dose-response relationship, Cox regression, melanoma, non-melanoma

## Abstract

Although hydrochlorothiazide (HCTZ) has been suggested to increase skin cancer risk in white Westerners, there is scant evidence for the same in Asians. We analyzed the association between the use of hydrochlorothiazide and non-melanoma in the Asian population using the common data model. Methods: A retrospective multicenter observational study was conducted using a distributed research network to analyze the effect of HCTZ on skin cancer from 2004 to 2018. We performed Cox regression to evaluate the effects by comparing the use of HCTZ with other antihypertensive drugs. All analyses were re-evaluated using matched data using the propensity score matching (PSM). Then, the overall effects were evaluated by combining results with the meta-analysis. Results: Positive associations were observed in the use of HCTZ with high cumulative dose for non-melanoma skin cancer (NMSC) in univariate analysis prior to the use of PSM. Some negative associations were observed in the use of low and medium cumulative doses. Conclusion: Although many findings in our study were inconclusive, there was a non-significant association of a dose-response pattern with estimates increasing in cumulative dose of HCTZ. In particular, a trend with a non-significant positive association was observed with the high cumulative dose of HCTZ.

## 1. Introduction

Hypertension is a major risk factor for non-communicable diseases (NCDs), such as cardiovascular and renal diseases [1,2]. According to the World Health Organization (WHO), approximately over 20% of the global population suffered from hypertension in 2015. Traditionally, a person with consistent blood pressure (BP), exceeding 140/90 mmHg, had been diagnosed with hypertension. However, in 2017, the American College of Cardiology and the American Heart Association proposed a new BP threshold, 130/80 mmHg, to diagnose hypertension [3]. By adopting new BP thresholds, BP therapy would be recommended to millions of additional US adults [4], but it was expected to bring more benefits for society and individuals by reducing the risk of cardiovascular and cardiovascular-related diseases with interventions in an earlier course of progression [5,6,7]. However, after a new diagnosis, most hypertensive patients inevitably require more antihypertensive classes to achieve intensified BP target goals [8,9].

Antihypertensive pharmacotherapy is recommended to most hypertensive patients, due to the limited efficacy of lifestyle modifications, especially for high002Drisk hypertensive patients. Thiazide or thiazide-like diuretics are considered as one of the first-line classes in antihypertensive pharmacotherapy. Moreover, they are essential in combination therapy as volume and sodium retention is the main mechanism in treatment-resistant hypertension [10,11,12]. The diuretic hydrochlorothiazide (HCTZ) has been one of the most frequently prescribed antihypertensive drugs in the United States and Europe [13]. However, recent studies [14,15,16] have found that the use of HCTZ may potentially cause skin cancer due to the photosensitizing properties in thiazide diuretics [17]. On the contrary, Gandini et al. reported in their meta-analysis that they found no association between thiazide diuretics and skin cancer [18]. These inconsistent and uncertain observations between studies emphasize the challenges we face when interpreting the findings in real-world observational studies. In fact, despite the increased risk of skin cancer associated with the use of HCTZ in white Westerners, there is no evidence that the carcinogenic effect of HCTZ is substantial in the Asian population [19], whose incidence of skin cancer is much lower [20]. Therefore, to elucidate the association between the use of HCTZ and skin cancer clearly, a nationwide observational study with qualified assessment and fewer biases should be conducted through a distributed data system with standardized protocol.

In this study, we investigated the association between HCTZ and skin cancer, including melanoma and non-melanoma skin cancer (NMSC) by using the Observational Medical Outcomes Partnership (OMOP) Common Data Model (CDM)-based distributed research network. By processing all analyses in the CDM network with a standardized code, we also validated the utility of the CDM network for quick and reliable big data-based clinical observational research examining the potential carcinogenic risk of drugs.

## 2. Experimental Section

### 2.1. Data Sources

We conducted a retrospective cohort study with Korean patients visited in 1 of 3 hospitals (Seoul National University Hospital, Seoul National University Bundang Hospital, and Asan Medical Center) from 1 January 2004 to 28 February 2018 using Observational Health Data Sciences and Informatics open-source software and the OMOP CDM version 5.2 database. This study was approved with waiver of informed consent by the institutional review board (E-2002-017-1098).

### 2.2. Patient Selection

Our cohort consisted of patients aged 20–80 years who had a treatment history with HCTZ, other antihypertensive drugs, or both. We excluded participants who took immunosuppressive agents (azathioprine, cyclosporine, tacrolimus, or mycophenolate mofetil), had a medical history of HIV or organ transplant, and had previous melanoma or NMSC before taking antihypertensive drugs (including HCTZ). Considering reasonable exposure time to outcomes, we also excluded participants who had an outcome date within a month from the first date of taking any antihypertensive drugs. Then, we defined the group of any use of HCTZ as exposed, and the groups of other antihypertension medicine users (“Never use”) served as our reference. Furthermore, participants who underwent the HCTZ treatment were divided into 3 different groups: “HCTZ-only use” (no other prescriptions drugs than HCTZ) group, “Combination use” (use of combination of HCTZ and other hypertension diuretics) group, and “Ever use” (both HCTZ-only and Combination use) group.

### 2.3. Outcomes and Other Covariates

We identified melanoma skin cancer (C43, D03) and NMSC (C44, D04) with the Korean Standard Classification of Disease 8th revision (KCD-8) codes, which are similar to 10th revision of International Classification of Diseases (ICD-10) codes if available. We considered the first type of occurring skin cancer as the outcome of the study, so that each patient could contribute only once. Potential confounders were selected based on data available in the CDM network, and they were reviewed by clinicians; selected confounders were sex, age, use of drugs potentially affecting the risk of skin cancer (aspirin, nonsteroidal anti-inflammatory drug [NSAIDs], and statins) [21,22,23], history of diabetes, history of chronic obstructive pulmonary disease (COPD), and comorbidities using Charlson Comorbidity Index (CCI) scores [24] [categorized as 0, 1 and ≥2: a maximum count of score occurred]. History of diabetes and COPD were defined by any appearance of an ICD code or use of disease-specific drugs as Pedersen et al. detailed in [15]. A detailed list of concept ID and ICD codes, used for constructing target definitions and exclusion criteria, are provided in Supplementary Table S1.

### 2.4. Statistical Analysis

The association between HCTZ and skin cancer was investigated by using one standardized analytic code as the database of each hospital was converted using the format of OMOP CDM, a set of uniform data standards and formatting. We computed hazard ratios (HRs) with a Cox regression model to analyze the association between skin cancer and use of HCTZ. Additional analyses were performed to examine a potential dose-response relationship, and HRs were calculated for each group (“HCTZ-only, Combination, and Ever use”) based on the tertile levels in the cumulative HCTZ dose based on “HCTZ-only use.” Detailed information about the cut-off points of cumulative dose is described in Supplementary Table S2.

All analyses were conducted using univariate analysis and multivariate analysis adjusted with selected potential confounders. We performed the propensity score matching method (PSM) to control for selection bias by adjusting for the impact of given confounding factors [25]. All analyses were re-evaluated after checking for balance in the characteristics of matched groups. The overall effects were evaluated using the meta-analysis method, which is a statistical analysis combining the results of multiple studies. Statistical approaches (Cochran’s Q test and I^2^ value test) were conducted to test for heterogeneity in meta-analysis [26].

All analyses were performed using R 3.6.2 (http://www.R-project.org). Two-sided *p*-value less than 0.05 was considered to be statistically significant.

## 3. Results

### 3.1. Clinical Characteristics

**Figure 1:**
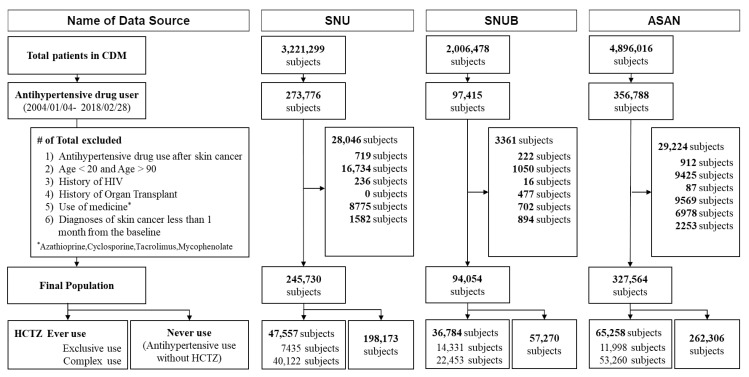
The flowchart of study participants in the CDM network.

Figure 1 illustrates the patient flowchart. After exclusions (*n* = 60,631), the total population from all 3 hospitals included in the study was 667,348 (149,599 HCTZ-user and 149,599 non-HCTZ-user). During 4,777,399 person-years, 1162 NMSC cases (1355 cases of total skin cancer) were observed in this study. Detailed information about the number of cases from each hospital are described in Table 1.

The descriptive characteristics of the exposure (“Ever use”) and non-exposure group (“Never use”) from each hospital before and after the PSM are described in Table 2. Patients in “Ever use” of HCTZ were older and had a higher comorbidity level compared to “Never use” of HCTZ. Also, the proportion of those who had a disease history (diabetes and COPD) and took medications (aspirin, NSAIDs, and statins) was higher than that of participants in “Never use” of HCTZ. However, those with use of only HCTZ (“HCTZ-only use”) had a lower high comorbidity level and had a lower proportion of disease history (COPD) and drug use (aspirin and statins) than patients with no prescription use of HCTZ (Supplementary Table S3).

### 3.2. Risk of NMSC Associated with HCTZ Use

**Figure 2:**
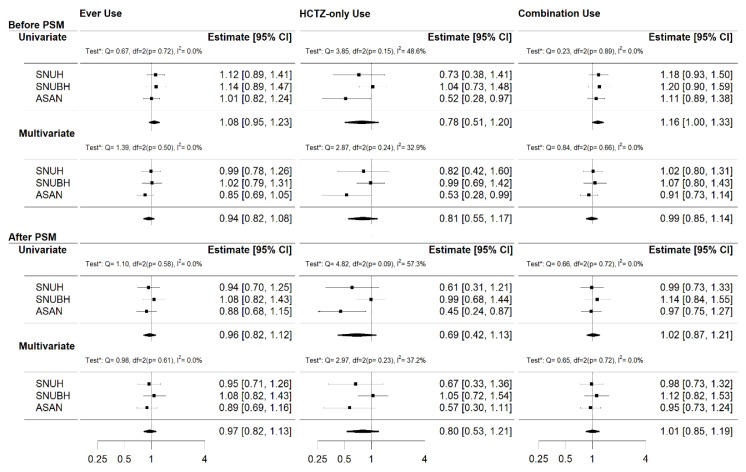
The overall effect of HCTZ use on NMSC in the CDM network for three cases (“Ever use”, “HCTZ-only use”, and “Combination use”). Each analysis was tested for heterogeneity (“Test*” indicates Cochran’s Q and I^2^ value tests). In the significant association, the chi-square test for heterogeneity was non-significant and its I^2^ value was zero. These suggest that there was little between-study variability in the found association.

Figure 2 presents the hazard ratios (HR) of HCTZ for the NMSC obtained from the meta-analysis. Before the PSM, the risk of NMSC was positively associated with the “Combination use” group in the univariate analysis (HR 1.16 [95% confidence interval, CI]; 1.00–1.33]). There was no evidence of statistical heterogeneity (*p* = 0.89, I^2^ = 0.0%). However, after PSM, no significant associations were observed.

**Figure 3:**
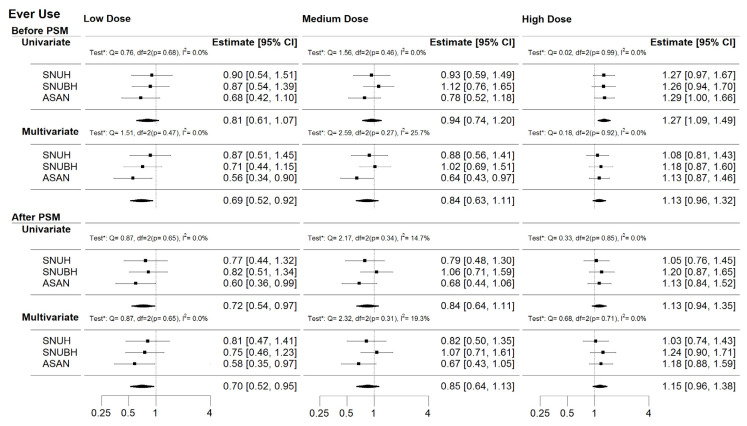
The overall dose-response effect of HCTZ use on NMSC in the CDM network for “Ever use”. Each analysis was tested for heterogeneity (“Test*” indicates Cochran’s Q and I^2^ value tests). In the significant associations, the chi-square tests for heterogeneity in most cases were insignificant and its I^2^ values were almost zero. These suggest that there was little between-study variability in the found associations.

In the dose-response analysis (Figure 3), the low cumulative dose of HCTZ in “Ever use” was negatively associated with the risk of NMSC in multivariate analysis before PSM (adjusted HR [aHR] 0.69 [95% CI; 0.52–0.92]) and in univariate (aHR 0.72 [95% CI; 0.54–0.97]) and multivariate analysis (aHR 0.70 [95% CI; 0.52–0.95]) after PSM. In contrast, the high cumulative dose of HCTZ in “Ever use” was positively associated with the risk of NMSC (HR 1.27 [95% CI 1.09–1.49]) and in “Combination use” (HR 1.28 [95% CI 1.08–1.51]; Supplementary Figure S1) only in the univariate analysis before PSM. In these significant associations, the chi-square tests for heterogeneity were insignificant and its I^2^ values were zeros. Although, there were no other significant associations, there was some evidence of a dose-response pattern with estimates increasing in cumulative dose of HCTZ with higher HRs in all the groups.

### 3.3. Additional Analyses

Additional analyses have been performed with melanoma and total skin cancer cases. The relation of exposure to HCTZ and the risk of each case was illustrated in Supplemental Figures S2 and S3. For the melanoma case (Supplemental Figure S2), analyses were hindered by a low number of cases: 183 cases in a total of 667,348 patients (2.5%). There was no evidence found that either HCTZ use or cumulative use of HCTZ were associated with the risk of the melanoma cancer in the univariate and multivariate analysis before and after PSM.

For total skin cancer (Supplementary Figure S3), the similar associations found in NMSC were observed. The risk of total skin cancer was positively associated with the “Combination use” group in the univariate analysis before PSM (HR 1.16 [95% CI 1.02–1.32]). The high cumulative dose of HCTZ in “Combination use” and “Ever use” had a positive association with risk only in the univariate analysis before PSM (HR 1.25 [95% CI; 1.07–1.47] and HR 1.25 [95% CI; 1.08–1.45]). The risk of total skin cancer was negatively associated with medium doses of HCTZ in “HCTZ-only use” in the univariate analysis after PSM (HR 0.54 [95% CI 0.35–0.96]). Other negative associations were observed in the low dose of HCTZ in “Ever use” in the multivariate analysis before, and after, PSM (HR 0.74 [95% CI; 0.57–0.96] and HR 0.74 [95% CI; 0.56–0.97]). A tendency toward an increased risk for the high cumulative use of HCTZ was observed in all groups with limited statistical significance.

## 4. Discussion

Our results showed limited evidence that the use of HCTZ in the Asian population was associated with a risk of NMSC. In the meta-analysis using CDM data of 3 hospitals enrolling 667,348 patients and observation for 4,777,399 person-years, no significant positive associations between HCTZ use, and the risk of NMSC were found after PSM adjusting for potential confounders (age, sex, use of aspirin, use of statins, use of NSAIDs, history of diabetes, history of COPD, and CCI scores). After PSM, some negative associations were observed in medium (“HCTZ-only use”) in the univariate analysis and low cumulative dose of HCTZ of “Ever use” in the multivariate analysis. Similar associations were observed for total skin cancer. Although many findings in our study were inconclusive, we observed an increased risk of NMSC for the high cumulative use of HCTZ in “Combination and Ever use” in all of the analyses.

A positive association between the use of HCTZ and an increased risk of skin cancer was reported in European studies, which utilized Danish nationwide health registries [14,15,16]. On the other hand, Gandini et al. have reported that they could find no association between thiazide diuretic use, and the risk of skin cancer in their meta-analysis [18]. Recently, a few studies were also conducted to clarify this association in Asia. From a nationwide cohort study conducted in South Korea, the protective effect of HCTZ has not observed the development of melanoma and non-melanoma skin cancer [27]. However, from another nationwide cohort study conducted in Taiwan, Pottegård et al. have reached limited evidence of HCTZ use associated with an increased risk of skin cancer [19]. Considering the minimal difference in racial and ethnic characteristics, the inconsistent results between South Korea and Taiwan may be due to the different methodological and analytical approaches between the studies. In this study, the protective effects in the medium cumulative use of HCTZ with other hypertensive drugs were observed only for NMSC, but the dose amounts were relatively smaller(<~5400 mg) than other those in other European studies. Although, there was limited evidence to clarify the association, a trend of increased risk of skin cancer was observed with an increased dose of HCTZ in “Combination and Ever use” in all analyses. As a previous study reported that thiazide diuretics were the only class of photosensitizing antihypertensive drugs attained statistical significance related to an increased risk in skin cancer [17], our study results implicitly support that any cumulative thiazide diuretics use (in HCTZ only or combination) should be used with caution.

Recently, clinicians have been advised to reduce the use of HCTZ or prescribe HCTZ with caution. The Medicines and Healthcare products Regulatory Agency (MHRA) has stated the risk of the use of HCTZ-containing products and recommended to advise patients about the risk of non-melanoma skin cancer. The Ministry of Food and Drug Safety in Korea also recommended that clinicians reduce the use of HCTZ-containing products in clinical practice. However, the discontinued use of HCTZ may not be possible unless an equally effective alternative to HCTZ is found for hypertension. More studies with various approaches will be conducted to clarify this association, as different results can be observed, due to several factors, including different ethnicities, sample sizes, inconsistent definitions, inconsistent confounders, etc.

In a review paper, Kreutz et al. pointed out that “the overall inconsistent and puzzling results on the risk of skin cancer” is an example of the difficulties in observational studies that make a clear conclusion for clinical practice. The authors added that the carcinogenic risk of drugs may only be examined through observational studies due to the difficulties of randomized clinical trials [28]. A well-designed observational study needs to be conducted with a solid and consistent methodology to reduce biases, and our current study has explored the possibility to design such an observational study using the CDM network.

The main strength of the current study was the standardized analysis and coding algorithm for multicenter data sources fitted for global and large-scale observational research. In order to examine the association between skin cancer and HCTZ use, we used a single standardized analytics code to generate a summarized result from each of the three different data sources in the CDM network, and used the meta-analysis method to combine these results. Since there was no information other than the summarized results shared through the CDM network, our study excluded almost all the possible ways in which data could be traced back to individuals. Therefore, this study has proven that the acceleration and duplication of research at the national level may be possible [29], while minimizing inconsistent results caused by various methodological definitions and the risk of privacy leaks and data safety through a distributed research network [30].

Still, several limitations existed in this study. Although the data of three major hospitals were merged, thereby enrolling a total of 667,348 patients with a total of 4,777,399 person-years in the study, most of our findings were inconclusive. This may be explained by the low prevalence of skin cancer in Korea, particularly melanoma cases. Compared to Western countries, the prevalence of skin cancer in South Korea is relatively low. Moreover, the ratio of the basal cell carcinoma (BCC) and squamous cell carcinoma (SCC), two major types of NMSC, may confound our results. In our study population, within “Ever use” at SNU hospital, the number of BCC (61 cases) is about 2.3 times over the SCC (26 cases). The low proportion of SCC, which has a significant association with HCTZ [31], may contribute to our inconclusive results. Our observations in the dose-response pattern with estimates increasing in cumulative dose of HCTZ may be reasonable, since Xavier et al. reported that the risk of BCC and/or SCC associated higher HCTZ dose [32]. A more concise and clear conclusion is expected to be drawn when more data are standardized and available in the CDM network. In fact, because transferring electronic health records to the CDM network was still in progress, misclassifications of definitions (drugs, outcomes, and comorbidities) may exist in this study. We created our own mapping table to reduce such misclassifications by reviewing them with nurses and technicians.

## 5. Conclusions

Although there were no significant positive associations after PSM, there was some evidence of a dose-response pattern with estimates increasing in cumulative dose of HCTZ.

## Figures and Tables

**Table 1:** Number of Skin Cancer Cases in the Study Participants before and after Propensity Score Matching.

Center	PSM	Case	Subjects	Skin Cancer	Incident Density *	NMSC	Incident Density *	MSC	Incident Density *
SNU	Before	Never use	198,173	366	27.1	296	21.9	70	5.2
		Ever use ^1^	47,557	119	30.3	97	24.7	22	5.6
		HCTZ-only use ^2^	7435	12	21.4	9	16.0	3	5.4
	After	Never use	47,557	107	31.3	89	26.1	18	5.3
		Ever use ^1^	47,557	119	30.3	97	24.7	22	5.6
		HCTZ-only use ^2^	7435	12	21.4	9	16.0	3	5.4
SNUB	Before	Never use	57,270	134	39.3	124	36.4	10	2.9
		Ever use ^1^	36,784	127	44.2	120	41.7	7	2.4
		HCTZ-only use ^2^	14,331	42	38.3	41	37.4	1	0.9
	After	Never use	36,784	93	42.0	85	38.4	8	3.6
		Ever use ^1^	36,784	127	44.2	120	41.7	7	2.4
		HCTZ-only use ^2^	14,331	42	38.3	41	37.4	1	0.9
ASAN	Before	Never use	262,306	458	24.2	413	21.8	45	2.4
		Ever use ^1^	65,258	131	25.6	112	21.9	19	3.7
		HCTZ-only use ^2^	11,998	16	18.0	10	11.2	6	6.8
	After	Never use	36,784	93	42.0	85	38.4	8	3.6
		Ever use ^1^	36,784	127	44.2	120	41.7	7	2.4
		HCTZ-only use ^2^	14,331	42	38.3	41	37.4	1	0.9

PSM, propensity score matching; SNU, Seoul National University Hospital; SNUB, Seoul National University Bundang Hospital; ASAN, Asan Medical Center; NMSC, Non-melanoma skin cancer; MSC, Melanoma skin cancer. * per 100,000 person-years; ^1^ “Ever use” were those who have ever taken medication containing thiazide at least once; ^2^ “HCTZ-only use” were those who took thiazide alone without other antihypertensive medications.

**Table 2:** Study Participants’ Characteristics before and after Propensity Score Matching.

	SNU					
	Before Matching			After Matching		
	Never Use	Ever Use ^1^		Never Use	Ever Use ^1^	
	*N* (%)	*N* (%)	SMD ^2^	*N* (%)	*N* (%)	SMD ^2^
Age, Mean (SD)	50.60 (16.0)	51.36 (15.7)	0.048	51.35 (15.6)	51.36 (15.7)	<0.001
Female	112,452 (56.7)	27,111 (57.0)	0.005	27,275 (57.4)	27,111 (57.0)	0.007
CCI			0.271			0.008
0	84,327 (42.6)	15,553 (32.7)		15,375 (32.3)	15,553 (32.7)	
1	68,293 (34.5)	15,618 (32.8)		15,666 (32.9)	15,618 (32.8)	
≥2	45,553 (23.0)	16,386 (34.5)		16,516 (34.7)	16,386 (34.5)	
Disease history						
Diabetes	43,360 (21.9)	17,911 (37.7)	0.350	17,901 (37.6)	17,911 (37.7)	<0.001
COPD	13,266 (6.7)	5070 (10.7)	0.141	4747 (10.0)	5070 (10.7)	0.022
Drug use						
Aspirin	50,255 (25.4)	22,542 (47.4)	0.471	22,573 (47.5)	22,542 (47.4)	0.001
NSAIDS	70,329 (35.5)	17,181 (36.1)	0.013	16,831 (35.4)	17,181 (36.1)	0.015
Statins	52,885 (26.7)	21,434 (45.1)	0.391	21,535 (45.3)	21,434 (45.1)	0.004
	**ASAN**					
Age, Mean (SD)	59.15 (13.13)	62.66 (11.78)	0.281	62.69 (11.73)	62.66 (11.78)	0.003
Female	119,243 (45.5)	32,207 (49.4)	0.078	32,177 (49.3)	32,207 (49.4)	0.001
CCI			0.169			0.004
0	99,225 (37.8)	19,793 (30.3)		19,684 (30.2)	19,793 (30.3)	
1	73,747 (28.1)	18,962 (29.1)		18,947 (29.0)	18,962 (29.1)	
≥2	89,334 (34.1)	26,503 (40.6)		26,627 (40.8)	26,503 (40.6)	
Disease history						
Diabetes	65,625 (25.0)	21,033 (32.2)	0.160	21,076 (32.3)	21,033 (32.2)	0.001
COPD	18,120 (6.9)	5831 (8.9)	0.075	5583 (8.6)	5831 (8.9)	0.013
Drug use						
Aspirin	79,195 (30.2)	24,217 (37.1)	0.147	24,075 (36.9)	24,217 (37.1)	0.005
NSAIDS	100,020 (38.1)	33,107 (50.7)	0.256	33,064 (50.7)	33,107 (50.7)	0.001
Statins	89,715 (34.2)	27,593 (42.3)	0.167	27,615 (42.3)	27,593 (42.3)	0.001
	**SNUB**					
Age, Mean (SD)	61.37 (13.69)	63.43 (13.07)	0.154	63.52 (13.18)	63.43 (13.07)	0.007
Female	25,276 (44.1)	19,379 (52.7)	0.172	19,506 (53.0)	19,379 (52.7)	0.007
CCI			0.095			0.019
0	18,669 (32.6)	11,760 (32.0)		12,066 (32.8)	11,760 (32.0)	
1	21,802 (38.1)	12,713 (34.6)		12,637 (34.4)	12,713 (34.6)	
≥2	16,799 (29.3)	12,311 (33.5)		12,081 (32.8)	12,311 (33.5)	
Disease history						
Diabetes	16,872 (29.5)	11,467 (31.2)	0.037	11,174 (30.4)	11,467 (31.2)	0.017
COPD	2011 (3.5)	1459 (4.0)	0.024	1308 (3.6)	1459 (4.0)	0.022
Drug use						
Aspirin	27,751 (48.5)	19,150 (52.1)	0.072	18,773 (51.0)	19,150 (52.1)	0.021
NSAIDS	4300 (7.5)	3347 (9.1)	0.058	2972 (8.1)	3347 (9.1)	0.036
Statins	13,666 (23.9)	8420 (22.9)	0.023	8078 (22.0)	8420 (22.9)	0.022

CCI, Charlson Comorbidity Index; COPD, Chronic obstructive pulmonary disease; SNU, Seoul National University Hospital; SNUB, Seoul National University Bundang Hospital; ASAN, Asan Medical Center. ^1^ “Ever use” were those who have ever taken medication containing thiazide at least once; ^2^ SMD (Standardized mean differences) for all pairwise comparisons.

## Supplementary Materials

The following are available online at https://www.mdpi.com/2077-0383/9/9/2910/s1, Figure S1: The overall dose-response effect of HCTZ use on NMSC in the CDM network for “HCTZ-only use” and “Combination use.” Figure S2: The overall effect (a) and dose-response effect (b) of HCTZ use on melanoma skin cancer in the CDM network for three cases (“Ever use”, “HCTZ-only use”, and “Combination use”). Figure S3: The overall effect (a) and dose-response effect (b) of HCTZ use on total skin cancer in the CDM network for three cases (“Ever use”, “HCTZ-only use”, and “Combination use”). Table S1: Codes and definitions. Table S2: Tertile cut-off points of cumulative dose of HCTZ. Table S3: Study participants’ characteristics of HCTZ-only use before and after Propensity Score Matching.
