# Supplementary material for: Association between Use of Hydrochlorothiazide and Nonmelanoma Skin Cancer: Common Data Model Cohort Study in Asian Population
Source: J Clin Med. 2020 Sep 9;9(9):2910. doi: 10.3390/jcm9092910 (PMC7563303; doi:10.3390/jcm9092910)
Supplement: Supplementary file 1 [file jcm-09-02910-s001.pdf]

Supplementary Table 1. Codes and definitions

	ICD code/KCD code
**Melanoma**	C43,D03
**Non-Melanoma**	C44,D04
**HIV**	B20-24
	Z21
**Transplant**	Z94
**Diabetes**	E10-E14
**COPD**	J42-J44
**Charlson Comorbidity Index**	
Myocardial Infarction	I21-I23
Congestive Heart Failure	I50,I110,I130,I132
Periphral Vascular Disease	I70-74, I77
Cerebrovascular disease	I60-69, G45-46
Dementia	F00-03, F051, G30
Chronic pulmonary disease	J40-47, J60-67,J684, J701, J703,J841,J920,J961,J982,J983
Connective tissue disease	M05,M06,M08,M09,M30,M31,M32,M33,M34,M35,M36,D86
Ulcer disease	K25-28, K221
Mild liver disease	B18, K71,K73,K74,K76, K700-703 , K709
Diabetes mellitus	E100,E101,E109,E110,E111,E119
Hemiplegia	G81-G82
Moderate/severe renal disease	N00-07,N11,N14,N17-19,Q61,I12,I13
Diabetes mellitus with chronic complications	E102-108,E112-E118
Any tumor,except C43 C44	C00-42, C45-75
Leukemia	C91-95
Lymphoma	C81-85, C88,C90,C96
Moderate/severe liver disease	K72, K704,K766 , I85, B150, B160, B162, B190, K704, K766
Metastatic solid tumor	C76-80
AIDS [removed =HIV exclusion]	B20-22 , B24

		Concept ID for Drug
**Hypertensive drugs**	SNUH, SNUBH	1308851,1314008,1314614,1318859,1326014,1328689,1332419,1332525,1332527, 1334461,1334492,1340161,1351559,1351583,1351587,1353816,1353817,19011443, 19011549,19015804,19017656,19018811,19019238,19022242,19022948,19022949, 19028935,19028936,19073093,19073094,19074672,19074673,19076924,19096677, 19096678,19096740,19096752,19101573,19101751,19101798,19101807,19101835, 19102106,19102170,19102171,19104027,19106542,19106543,19106593,19106594, 19107812,19127432,19127433,19127434,19133570,19133621,21030573,21141332, 35603869,36883910,40022801,40058099,40162867,40162878,40163271,40163275, 40163312,40163342,40165082,40165762,40165789,40166187,40166188,40167202, 40167838,40167843,40167849,40167852,40171905,40171917,40173995,40185276, 40185304,40990862,41080575,41178428,42800549,43749671
	ASANH	42929942,42950402,21062424,40167844,42932556,1332529,42930491,1332442, 42929955,42929933,42955567,42955565,42950369,42950439,21101680,21160716, 40167850,40167853,42932934,1332531,42932720,21131351,42930635,42930549, 42930512,1332421,19018811,19012417,1314008,1314005,42800550,42942301, 42942303,41036326,43659489,43834200,42926025,42926012,42925993,1351588, 1351589,1351586,42957540,42957542,42944039,44034982,42948136,42950297, 42950296,42950286,42950287,42950301,42950290,42941377,42941384,36886059, 42941353,42941354,43191848,42953121,19068340,41179747,41220999,21095298, 1326014,42963292,42963310,19017657,21031868,40185277,40185304,40185305, 42944371,42944373,19025094,42963221,21084787,42963225,42952827,42935148, 42935150,42935143,19012420,42935145,42943253,42943254,21130963,21032918, 21023154,42938090,42938087,42936772,40163342,42938870,42938363,40728489, 40996076,42939160,43205730,43139742,42933405,1308877,42933281,1308876, 43268715,40171905,40171907,40171919,43785485,43605601,42960852,42960868, 42960727,42960736,42959788,42960000,42960006,42960003,42960009,42960028, 42960032,42960036,42960035,42948652,42948649,42948655,21070867,42963314, 42952814,21151257,21131491,19019331,43749671,41080575,40167838,42950292
**Hydrocholorothiazide drugs**	SNUH, SNUBH	974226,974473,974474,974642,974702,19023453,19023454,19078080,19078101,

		19078106,19102491,40184184,40184187,40184217,40224166,40224172,40224175
	ASANH	42925745,42925750,42950748,40044851,21102246,21023664,42950601,42955595,43288515,43293966,21072870,21092336,42950497,42938852,42960021,40165771,40044817,40044832,42938519
**Immunosuppressive agents** (azathioprine, cyclosporine, tacrolimus, mycophenolate)	SNUH, SNUBH	19079712, 35606029, 19076045, 40928406,19010551, 19022826,41115995,950662,19078956,19021102,19082899,950641,950669,41114885,950667,19010484,19014880
	ASANH	19014880,42966092,19007424,21039533,42921286,41356321,42919788,19082899,42947987,42947985,42947986,42948047,43271717,19010484,42948040,42948038,42948039,19010554,19010557,19010560,21118393,19004061,42956678,42956679,42956675,21082760,950664,42927613,21171169,19010270,42927625,21073062,43278387,43267426,42927622,42927644
**Use of Drug (Aspirin)**	SNUH	1112896, 1113143,1718409,19021575,42483115,42949808,42949815
	SNUBH	1112807, 1113348,43265705,1718409,1112896,42949808
	ASANH	43292896,42950014,42949963,1113143,42949962,19068100,19001045,1112896,42950105,42949895
**Use of Drug (NSAIDs)**	SNUH, SNUBH	1113672,1118091,1146847,1178665,1195496,1236610,1236611,19019050,19019071,19019273,19029024,19029025,19029394,19078461,19081237,19088915,19103397,19103398,19103697,19106551,19133853,21065513,40097538,40222578,40992339,41272174,46275899,46287424
	ASANH	42948831,1178665,19034015,41250488,42926462,42926625,21144042,41127443,1118087,1118088,1118113,42923464,19007821,21053002,19128678,19024591,19078461,43184525,45774493,42922470,42922476,42922469,42953301,19133853,40164851,42947240,43256386,42952574,43288947,42965811,1146847,19008125,42973718,42936726,42936728,42923089,21156633,42923602,42923612,40054158,19024626,41339644,40717541,40717539,42917412,42917663,42917162,42971817,42971818,42971822,40048787,19134398,19135542,36894840,19019272,42973730,40719017,42922582,41240436
**Use of Drug (Statins)**	SNUH, SNUBH	1539407,1539411,1545959,1545996,1545997,19019116,19077498,19086163,19123592,40165245,40165253,40165261,40165642,40165646,40175394,40175400
	ASANH	42972751,1545998,42972960,1545999,19123593,19068781,19003051,21035494,19019116,19031830,40165643,40165647,42970630,42969416,42969642,42969821,40165246,40165254,40165262,42936874,42936866,19004082,42936791,1539406,19003030,19004081,42970634,42970626,40744805
**Use of Diabetes drugs**	SNUH, SNUBH	1502829,1525221,1529352,1597758,1597761,1597772,1597773,1597781,1597792,19006931,19021312,19023424,19023425,19023426,19059797,19059800,19077682,19078552,19078558,19079293,19079465,19106521,19107110,19125041,19125045,19125049,19129179,21081251,21091002,21169719,35145801,35153532,35602725,36884883,36887702,36889815,40044222,40163928,40164891,40164897,40164922,40164929,40164943,40164946,40166037,40166041,40231394,40231402,40239218,42708086,42708090,42708168,42902468,42902742,42902821,42902992,42953698,42953818,42960587,42960590,42960593,42960599,42960642,42960645,42960648,42960653,42960773,42961319,42961322,42961325,42961331,42961484,42961487,42961490,42961494,42961500,42962884,42969162,43013896,43013899,43013911,43013915,43013918,43013924,43256461,43264421,43267262,43286269,43515533,44785831,45774436,45774445,45774754,45774893,45775456,45775624,46233971,46233977,46234050,46234097,46234234,46234239,46287680,46287689
	ASANH	21121903,21112205,19001408,19024508,42939570,21035582,1597757,42954018,1597760,21055250,43203430,43197104,43518609,19078530,1596960,19135263,36278491,19078551,42954469,1525216,42968760,42968762,19054443,19054444,19054445,42939568,40135391,40129534,19024503,40046201,42954472,42954464,

		1525217,40098705
**Use of COPD drugs**	SNUH, SNUBH	19112761,19134101,42873622,43169383,44025365
	ASANH	40727757,21048311,42920472,42480257,21138355

Supplementary Table 2. Tertile cut-off points of cumulative dose of HCTZ

**[DOSE]**	**[Low]**	**[Medium]**	**[High]**
	1st tertile	2nd tertile	3rd tertile
SNUH [mg]	[0 - 750.0]	[750.1 - 4900.0]	[>4900.1]
Mean±SD	286.1±236.1	2335.8±1189.3	29577.0±81869.5
SNUBH [mg]	[0 - 750.0]	[750.1 - 5362.5]	[>5362.6]
Mean±SD	315.1±234.0	2473.1±1302.4	24115.4±36172.3
ASAN [mg]	[0 - 250.0]	[250.1 - 1750.0]	[>1750.1]
Mean±SD	100.1±69.7	777.7±411.4	18071.6±21320.0

SNUH, Seoul National University Hospital; SNUBH, Seoul National University Bundang Hospital; ASAN, Asan Medical Center

Supplementary Table3. Study participants' characteristics of HCTZ-only use before and after Propensity Score Matching

	SNUH					
	Before Matching			After Matching		
	Never use	HCTZ-only use[1]		Never use	HCTZ-only use[1]	
	N (%)	N (%)	SMD[2]	N (%)	N (%)	SMD[2]
Age, Mean (SD)	50.60 (15.96)	50.45 (16.06)	0.009	50.39 (16.00)	50.45 (16.06)	0.003
Female	112,452 (56.7)	4,224 (56.8)	0.001	4,235 (57.0)	4,224 (56.8)	0.003
CCI			0.219			0.002
0	84,327 (42.6)	3,914 (52.6)		3,913 (52.6)	3,914 (52.6)	
1	68,293 (34.5)	2,304 (31.0)		2,301 (30.9)	2,304 (31.0)	
≥ 2	45,553 (23.0)	1,217 (16.4)		1,221 (16.4)	1,217 (16.4)	
Disease history						
Diabetes	43,360 (21.9)	1,545 (20.8)	0.027	1,549 (20.8)	1,545 (20.8)	0.001
COPD	13,266 (6.7)	290 (3.9)	0.125	287 (3.9)	290 (3.9)	0.002
Drug use						
Aspirin	50,255 (25.4)	1,422 (19.1)	0.15	1,421 (19.1)	1,422 (19.1)	<0.001
NSAIDS	70,329 (35.5)	1,776 (23.9)	0.256	1,778 (23.9)	1,776 (23.9)	0.001
Statins	52,885 (26.7)	1,533 (20.6)	0.143	1,533 (20.6)	1,533 (20.6)	<0.001
	ASAN					
Age, Mean (SD)	59.15 (13.13)	59.88 (13.00)	0.056	59.91 (12.97)	59.88 (13.00)	0.002
Female	119,243 (45.5)	6,769 (56.4)	0.221	6,764 (56.4)	6,769 (56.4)	0.001
CCI			0.201			0.001
0	99,225 (37.8)	5,315 (44.3)		5,308 (44.2)	5,315 (44.3)	
1	73,747 (28.1)	3,681 (30.7)		3,683 (30.7)	3,681 (30.7)	
≥ 2	89,334 (34.1)	3,002 (25.0)		3,007 (25.1)	3,002 (25.0)	
Disease history						
Diabetes	65,625 (25.0)	2,217 (18.5)	0.159	2,217 (18.5)	2,217 (18.5)	<0.001
COPD	18,120 (6.9)	540 (4.5)	0.104	517 (4.3)	540 (4.5)	0.009
Drug use						
Aspirin	79,195 (30.2)	1,569 (13.1)	0.425	1,577 (13.1)	1,569 (13.1)	0.002
NSAIDS	100,020 (38.1)	4,965 (41.4)	0.066	4,963 (41.4)	4,965 (41.4)	<0.001
Statins	89,715 (34.2)	2,339 (19.5)	0.337	2,341 (19.5)	2,339 (19.5)	<0.001
	SNUBH					
Age, Mean (SD)	61.37 (13.69)	61.85 (13.80)	0.035	61.77 (13.83)	61.85 (13.80)	0.006
Female	25,276 (44.1)	8,086 (56.4)	0.248	8,060 (56.2)	8,086 (56.4)	0.004
CCI			0.275			0.002
0	18,669 (32.6)	6,515 (45.5)		6,515 (45.5)	6,515 (45.5)	
1	21,802 (38.1)	4,754 (33.2)		4,765 (33.2)	4,754 (33.2)	
≥ 2	16,799 (29.3)	3,062 (21.4)		3,051 (21.3)	3,062 (21.4)	
Disease history						
Diabetes	16,872 (29.5)	3,147 (22.0)	0.172	3,112 (21.7)	3,147 (22.0)	0.006
COPD	2,011 (3.5)	397 (2.8)	0.043	348 (2.4)	397 (2.8)	0.021
Drug use						
Aspirin	27,751 (48.5)	4,953 (34.6)	0.285	4,961 (34.6)	4,953 (34.6)	0.001
NSAIDS	4,300 (7.5)	1,069 (7.5)	0.002	984 (6.9)	1,069 (7.5)	0.023

Statins	13,666 (23.9)	1,717 (12.0)	0.314	1,694 (11.8)	1,717 (12.0)	0.005

CCI, Charlson Comorbidity Index; COPD, Chronic obstructive pulmonary disease

1. "HCTZ-only use" were those who took thiazide alone without other antihypertensive medications.

2. SMD (Standardized mean differences) for all pairwise comparisons

Supplementary figure 1. The overall dose-response effect of HCTZ use on NMSC in the CDM network for "HCTZ-only use" and "Combination use." Each analysis was tested for heterogeneity ("Test*" indicates Cochran's Q and I² value tests). In the significant associations, the chi-square tests for heterogeneity in most cases were insignificant and its I² value were almost zero. These suggest that there was little between-study variability in the found association.

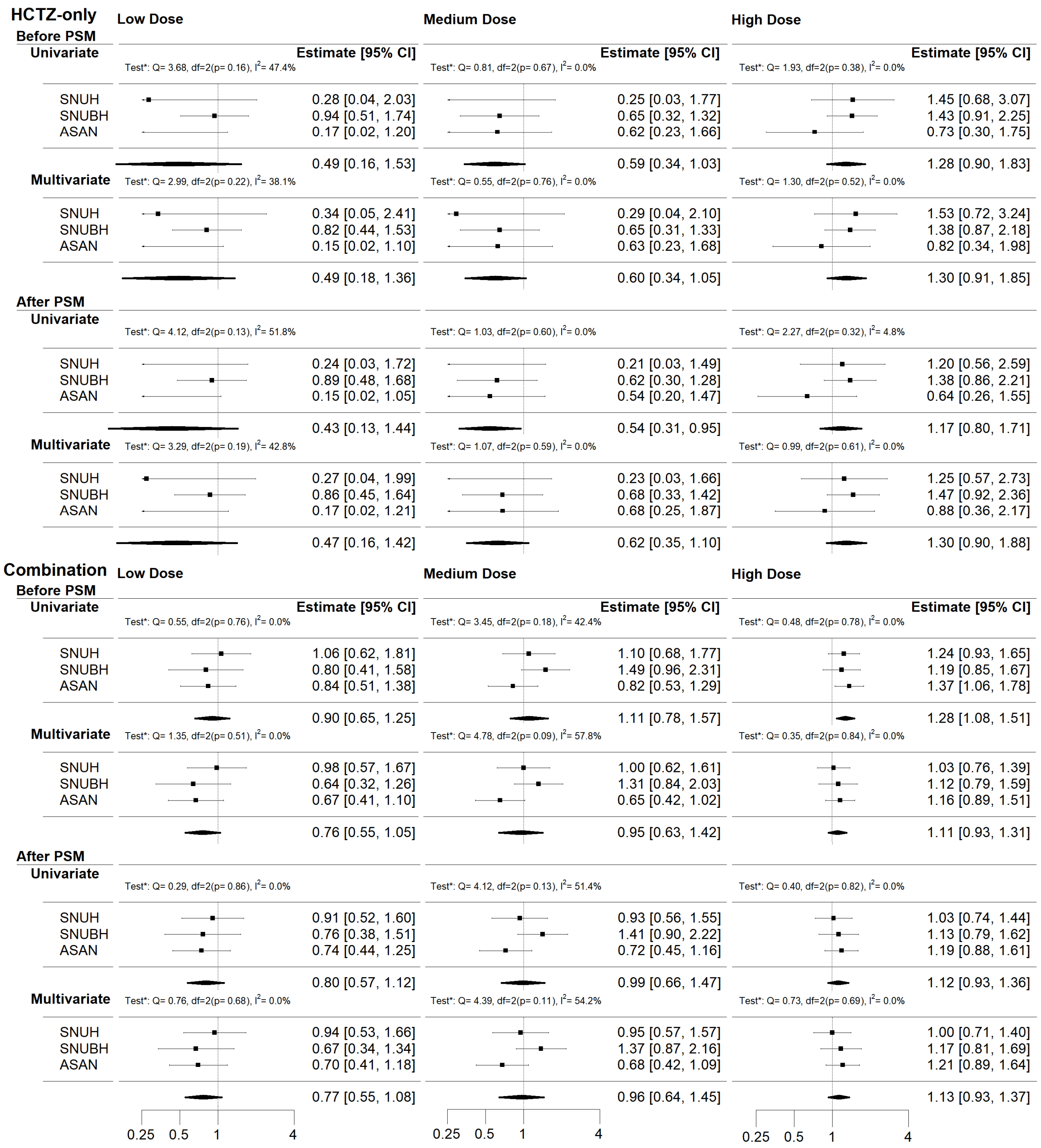

Supplementary figure 2. The overall effect (a) and dose-response effect (b) of HCTZ use on melanoma skin cancer in the CDM network for three cases ("Ever use", "HCTZ-only use", and "Combination use"). The dose-response effect in "HCTZ-only use" wasn't included since there wasn't enough cases for some tertiles. In these analyses, no associations were found in a statistically significance.

(a)

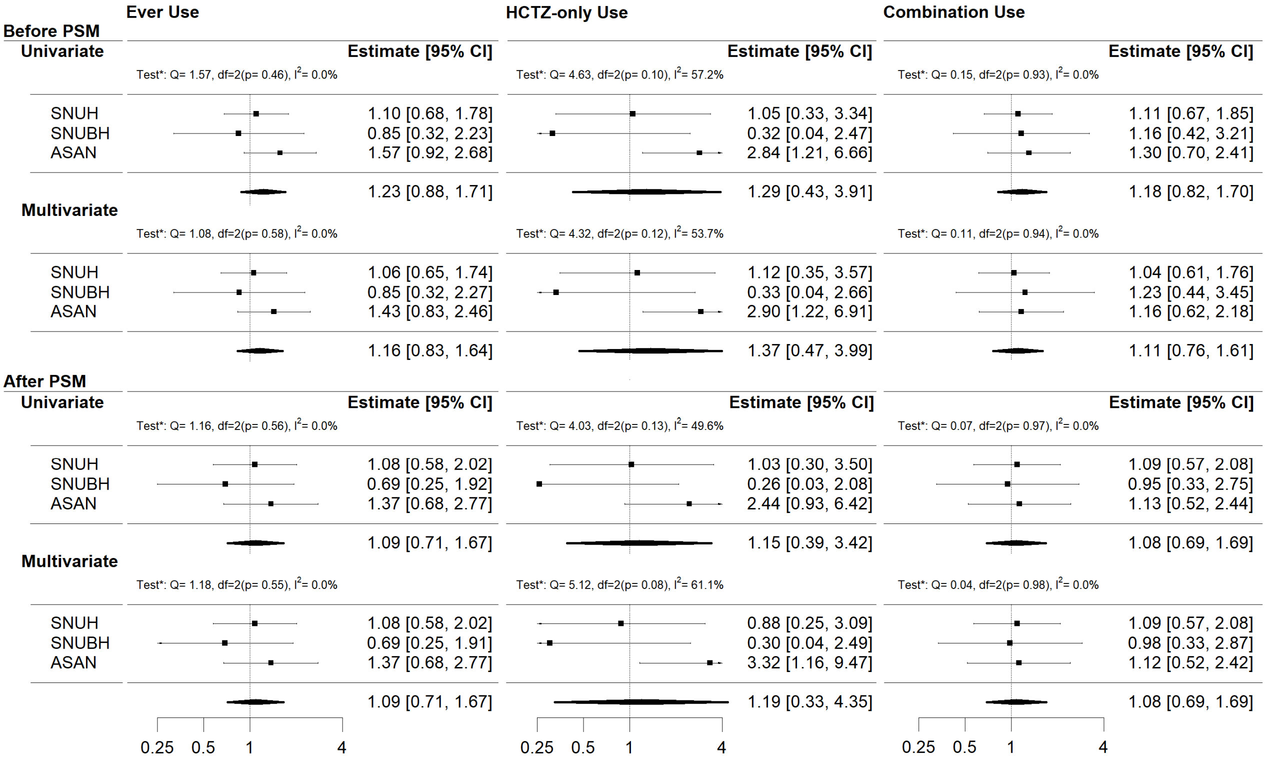

(b)

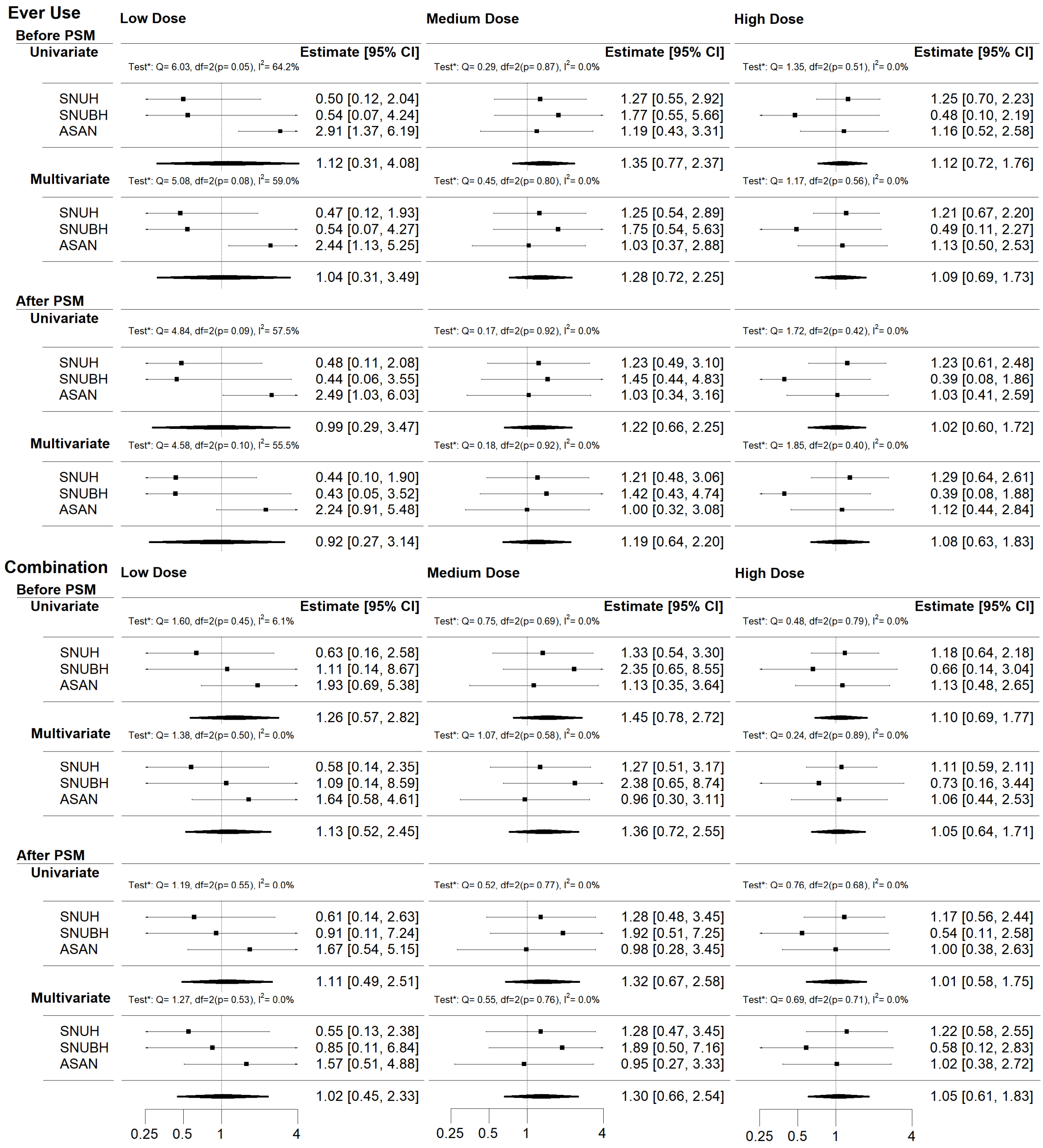
Ever Use
Low Dose
Medium Dose
High Dose
Before PSM
Univariate
Estimate [95% CI]
Test*: Q= 6.03, df=2(p= 0.05), I2= 64.2%
Test*: Q= 0.29, df=2(p= 0.87), I2= 0.0%
Test*: Q= 1.35, df=2(p= 0.51), I2= 0.0%
SNUH
SNUBH
ASAN
0.50 [0.12, 2.04]
0.54 [0.07, 4.24]
2.91 [1.37, 6.19]
1.12 [0.31, 4.08]
1.27 [0.55, 2.92]
1.77 [0.55, 5.66]
1.19 [0.43, 3.31]
1.35 [0.77, 2.37]
1.25 [0.70, 2.23]
0.48 [0.10, 2.19]
1.16 [0.52, 2.58]
1.12 [0.72, 1.76]
Multivariate
Test*: Q= 5.08, df=2(p= 0.08), I2= 59.0%
Test*: Q= 0.45, df=2(p= 0.80), I2= 0.0%
Test*: Q= 1.17, df=2(p= 0.56), I2= 0.0%
0.47 [0.12, 1.93]
0.54 [0.07, 4.27]
2.44 [1.13, 5.25]
1.04 [0.31, 3.49]
1.25 [0.54, 2.89]
1.75 [0.54, 5.63]
1.03 [0.37, 2.88]
1.28 [0.72, 2.25]
1.21 [0.67, 2.20]
0.49 [0.11, 2.27]
1.13 [0.50, 2.53]
1.09 [0.69, 1.73]
After PSM
Univariate
Test*: Q= 4.84, df=2(p= 0.09), I2= 57.5%
Test*: Q= 0.17, df=2(p= 0.92), I2= 0.0%
Test*: Q= 1.72, df=2(p= 0.42), I2= 0.0%
0.48 [0.11, 2.08]
0.44 [0.06, 3.55]
2.49 [1.03, 6.03]
0.99 [0.29, 3.47]
1.23 [0.49, 3.10]
1.45 [0.44, 4.83]
1.03 [0.34, 3.16]
1.22 [0.66, 2.25]
1.23 [0.61, 2.48]
0.39 [0.08, 1.86]
1.03 [0.41, 2.59]
1.02 [0.60, 1.72]
Multivariate
Test*: Q= 4.58, df=2(p= 0.10), I2= 55.5%
Test*: Q= 0.18, df=2(p= 0.92), I2= 0.0%
Test*: Q= 1.85, df=2(p= 0.40), I2= 0.0%
0.44 [0.10, 1.90]
0.43 [0.05, 3.52]
2.24 [0.91, 5.48]
0.92 [0.27, 3.14]
1.21 [0.48, 3.06]
1.42 [0.43, 4.74]
1.00 [0.32, 3.08]
1.19 [0.64, 2.20]
1.29 [0.64, 2.61]
0.39 [0.08, 1.88]
1.12 [0.44, 2.84]
1.08 [0.63, 1.83]
Combination
Low Dose
Medium Dose
High Dose
Before PSM
Univariate
Estimate [95% CI]
Test*: Q= 1.60, df=2(p= 0.45), I2= 6.1%
Test*: Q= 0.75, df=2(p= 0.69), I2= 0.0%
Test*: Q= 0.48, df=2(p= 0.79), I2= 0.0%
0.63 [0.16, 2.58]
1.11 [0.14, 8.67]
1.93 [0.69, 5.38]
1.26 [0.57, 2.82]
1.33 [0.54, 3.30]
2.35 [0.65, 8.55]
1.13 [0.35, 3.64]
1.45 [0.78, 2.72]
1.18 [0.64, 2.18]
0.66 [0.14, 3.04]
1.13 [0.48, 2.65]
1.10 [0.69, 1.77]
Multivariate
Test*: Q= 1.38, df=2(p= 0.50), I2= 0.0%
Test*: Q= 1.07, df=2(p= 0.58), I2= 0.0%
Test*: Q= 0.24, df=2(p= 0.89), I2= 0.0%
0.58 [0.14, 2.35]
1.09 [0.14, 8.59]
1.64 [0.58, 4.61]
1.13 [0.52, 2.45]
1.27 [0.51, 3.17]
2.38 [0.65, 8.74]
0.96 [0.30, 3.11]
1.36 [0.72, 2.55]
1.11 [0.59, 2.11]
0.73 [0.16, 3.44]
1.06 [0.44, 2.53]
1.05 [0.64, 1.71]
After PSM
Univariate
Test*: Q= 1.19, df=2(p= 0.55), I2= 0.0%
Test*: Q= 0.52, df=2(p= 0.77), I2= 0.0%
Test*: Q= 0.76, df=2(p= 0.68), I2= 0.0%
0.61 [0.14, 2.63]
0.91 [0.11, 7.24]
1.67 [0.54, 5.15]
1.11 [0.49, 2.51]
1.28 [0.48, 3.45]
1.92 [0.51, 7.25]
0.98 [0.28, 3.45]
1.32 [0.67, 2.58]
1.17 [0.56, 2.44]
0.54 [0.11, 2.58]
1.00 [0.38, 2.63]
1.01 [0.58, 1.75]
Multivariate
Test*: Q= 1.27, df=2(p= 0.53), I2= 0.0%
Test*: Q= 0.55, df=2(p= 0.76), I2= 0.0%
Test*: Q= 0.69, df=2(p= 0.71), I2= 0.0%
0.55 [0.13, 2.38]
0.85 [0.11, 6.84]
1.57 [0.51, 4.88]
1.02 [0.45, 2.33]
1.28 [0.47, 3.45]
1.89 [0.50, 7.16]
0.95 [0.27, 3.33]
1.30 [0.66, 2.54]
1.22 [0.58, 2.55]
0.58 [0.12, 2.83]
1.02 [0.38, 2.72]
1.05 [0.61, 1.83]
0.25
0.5
1
4

Supplementary figure3. The overall effect (a) and dose-response effect (b) of HCTZ use on total skin cancer in the CDM network for three cases ("Ever use", "HCTZ-only use", and "Combination use"). Each analysis was tested for the heterogeneity ("Test*" indicates Cochran's Q and I^2 value tests). In the significant associations, the chi-square test for heterogeneity was insignificant and its I^2 value was zero. These suggest that there was little between-study variability in the found association.

(a)

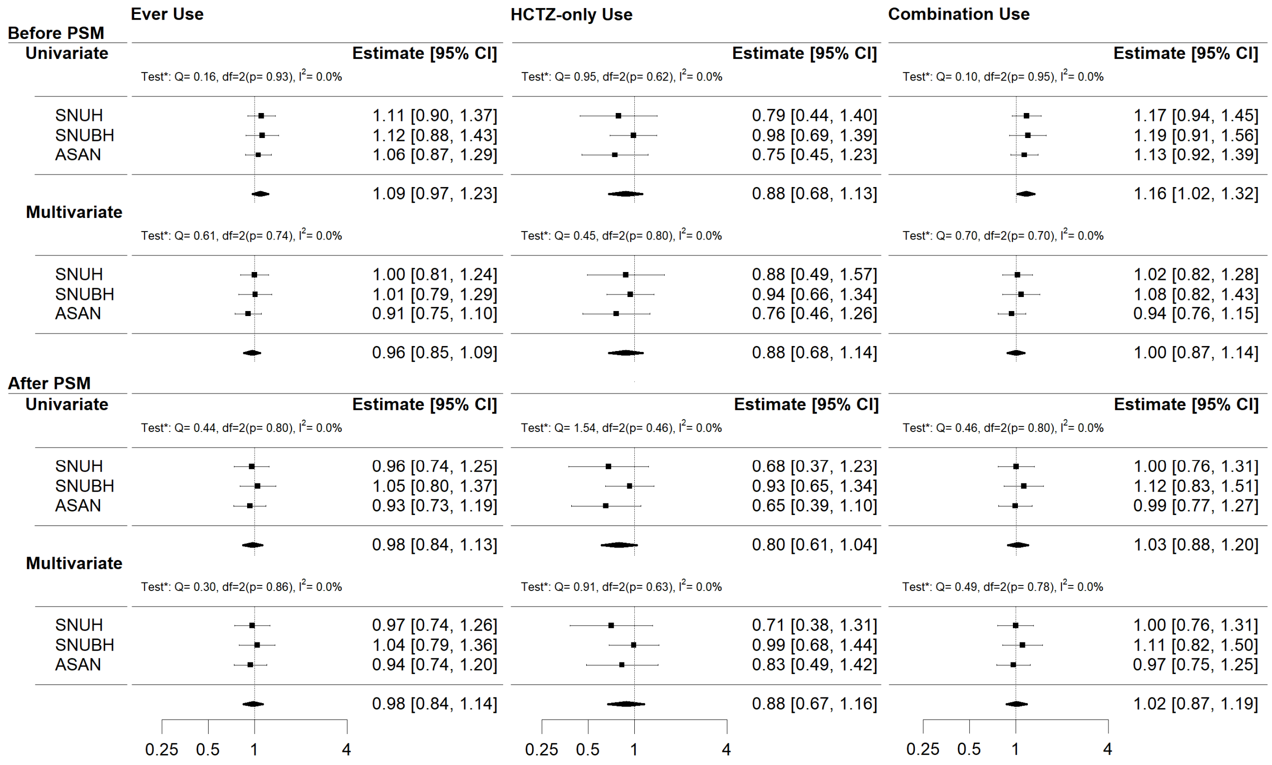

(b)

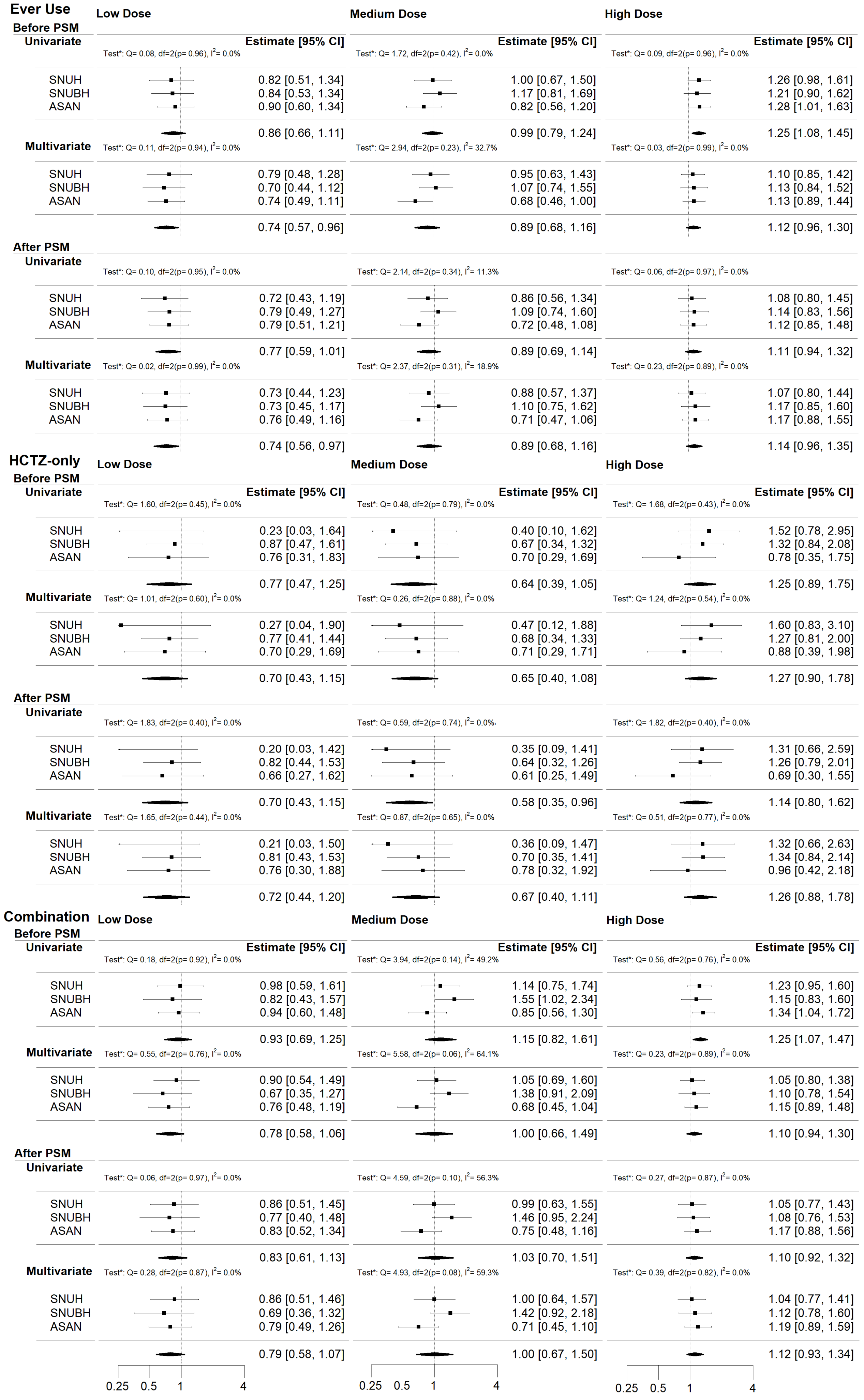
Ever Use
Low Dose
Medium Dose
High Dose
Before PSM
Univariate
Estimate [95% CI]
Test*: Q= 0.08, df=2(p= 0.96), I²= 0.0%
SNUH 0.82 [0.51, 1.34]
SNUBH 0.84 [0.53, 1.34]
ASAN 0.90 [0.60, 1.34]
0.86 [0.66, 1.11]
Test*: Q= 1.72, df=2(p= 0.42), I²= 0.0%
1.00 [0.67, 1.50]
1.17 [0.81, 1.69]
0.82 [0.56, 1.20]
0.99 [0.79, 1.24]
Test*: Q= 0.09, df=2(p= 0.96), I²= 0.0%
1.26 [0.98, 1.61]
1.21 [0.90, 1.62]
1.28 [1.01, 1.63]
1.25 [1.08, 1.45]
Multivariate
Test*: Q= 0.11, df=2(p= 0.94), I²= 0.0%
SNUH 0.79 [0.48, 1.28]
SNUBH 0.70 [0.44, 1.12]
ASAN 0.74 [0.49, 1.11]
0.74 [0.57, 0.96]
Test*: Q= 2.94, df=2(p= 0.23), I²= 32.7%
0.95 [0.63, 1.43]
1.07 [0.74, 1.55]
0.68 [0.46, 1.00]
0.89 [0.68, 1.16]
Test*: Q= 0.03, df=2(p= 0.99), I²= 0.0%
1.10 [0.85, 1.42]
1.13 [0.84, 1.52]
1.13 [0.89, 1.44]
1.12 [0.96, 1.30]
After PSM
Univariate
Test*: Q= 0.10, df=2(p= 0.95), I²= 0.0%
SNUH 0.72 [0.43, 1.19]
SNUBH 0.79 [0.49, 1.27]
ASAN 0.79 [0.51, 1.21]
0.77 [0.59, 1.01]
Test*: Q= 2.14, df=2(p= 0.34), I²= 11.3%
0.86 [0.56, 1.34]
1.09 [0.74, 1.60]
0.72 [0.48, 1.08]
0.89 [0.69, 1.14]
Test*: Q= 0.06, df=2(p= 0.97), I²= 0.0%
1.08 [0.80, 1.45]
1.14 [0.83, 1.56]
1.12 [0.85, 1.48]
1.11 [0.94, 1.32]
Multivariate
Test*: Q= 0.02, df=2(p= 0.99), I²= 0.0%
SNUH 0.73 [0.44, 1.23]
SNUBH 0.73 [0.45, 1.17]
ASAN 0.76 [0.49, 1.16]
0.74 [0.56, 0.97]
Test*: Q= 2.37, df=2(p= 0.31), I²= 18.9%
0.88 [0.57, 1.37]
1.10 [0.75, 1.62]
0.71 [0.47, 1.06]
0.89 [0.68, 1.16]
Test*: Q= 0.23, df=2(p= 0.89), I²= 0.0%
1.07 [0.80, 1.44]
1.17 [0.85, 1.60]
1.17 [0.88, 1.55]
1.14 [0.96, 1.35]
HCTZ-only
Low Dose
Medium Dose
High Dose
Before PSM
Univariate
Estimate [95% CI]
Test*: Q= 1.60, df=2(p= 0.45), I²= 0.0%
SNUH 0.23 [0.03, 1.64]
SNUBH 0.87 [0.47, 1.61]
ASAN 0.76 [0.31, 1.83]
0.77 [0.47, 1.25]
Test*: Q= 0.48, df=2(p= 0.79), I²= 0.0%
0.40 [0.10, 1.62]
0.67 [0.34, 1.32]
0.70 [0.29, 1.69]
0.64 [0.39, 1.05]
Test*: Q= 1.68, df=2(p= 0.43), I²= 0.0%
1.52 [0.78, 2.95]
1.32 [0.84, 2.08]
0.78 [0.35, 1.75]
1.25 [0.89, 1.75]
Multivariate
Test*: Q= 1.01, df=2(p= 0.60), I²= 0.0%
SNUH 0.27 [0.04, 1.90]
SNUBH 0.77 [0.41, 1.44]
ASAN 0.70 [0.29, 1.69]
0.70 [0.43, 1.15]
Test*: Q= 0.26, df=2(p= 0.88), I²= 0.0%
0.47 [0.12, 1.88]
0.68 [0.34, 1.33]
0.71 [0.29, 1.71]
0.65 [0.40, 1.08]
Test*: Q= 1.24, df=2(p= 0.54), I²= 0.0%
1.60 [0.83, 3.10]
1.27 [0.81, 2.00]
0.88 [0.39, 1.98]
1.27 [0.90, 1.78]
After PSM
Univariate
Test*: Q= 1.83, df=2(p= 0.40), I²= 0.0%
SNUH 0.20 [0.03, 1.42]
SNUBH 0.82 [0.44, 1.53]
ASAN 0.66 [0.27, 1.62]
0.70 [0.43, 1.15]
Test*: Q= 0.59, df=2(p= 0.74), I²= 0.0%
0.35 [0.09, 1.41]
0.64 [0.32, 1.26]
0.61 [0.25, 1.49]
0.58 [0.35, 0.96]
Test*: Q= 1.82, df=2(p= 0.40), I²= 0.0%
1.31 [0.66, 2.59]
1.26 [0.79, 2.01]
0.69 [0.30, 1.55]
1.14 [0.80, 1.62]
Multivariate
Test*: Q= 1.65, df=2(p= 0.44), I²= 0.0%
SNUH 0.21 [0.03, 1.50]
SNUBH 0.81 [0.43, 1.53]
ASAN 0.76 [0.30, 1.88]
0.72 [0.44, 1.20]
Test*: Q= 0.87, df=2(p= 0.65), I²= 0.0%
0.36 [0.09, 1.47]
0.70 [0.35, 1.41]
0.78 [0.32, 1.92]
0.67 [0.40, 1.11]
Test*: Q= 0.51, df=2(p= 0.77), I²= 0.0%
1.32 [0.66, 2.63]
1.34 [0.84, 2.14]
0.96 [0.42, 2.18]
1.26 [0.88, 1.78]
Combination
Low Dose
Medium Dose
High Dose
Before PSM
Univariate
Estimate [95% CI]
Test*: Q= 0.18, df=2(p= 0.92), I²= 0.0%
SNUH 0.98 [0.59, 1.61]
SNUBH 0.82 [0.43, 1.57]
ASAN 0.94 [0.60, 1.48]
0.93 [0.69, 1.25]
Test*: Q= 3.94, df=2(p= 0.14), I²= 49.2%
1.14 [0.75, 1.74]
1.55 [1.02, 2.34]
0.85 [0.56, 1.30]
1.15 [0.82, 1.61]
Test*: Q= 0.56, df=2(p= 0.76), I²= 0.0%
1.23 [0.95, 1.60]
1.15 [0.83, 1.60]
1.34 [1.04, 1.72]
1.25 [1.07, 1.47]
Multivariate
Test*: Q= 0.55, df=2(p= 0.76), I²= 0.0%
SNUH 0.90 [0.54, 1.49]
SNUBH 0.67 [0.35, 1.27]
ASAN 0.76 [0.48, 1.19]
0.78 [0.58, 1.06]
Test*: Q= 5.58, df=2(p= 0.06), I²= 64.1%
1.05 [0.69, 1.60]
1.38 [0.91, 2.09]
0.68 [0.45, 1.04]
1.00 [0.66, 1.49]
Test*: Q= 0.23, df=2(p= 0.89), I²= 0.0%
1.05 [0.80, 1.38]
1.10 [0.78, 1.54]
1.15 [0.89, 1.48]
1.10 [0.94, 1.30]
After PSM
Univariate
Test*: Q= 0.06, df=2(p= 0.97), I²= 0.0%
SNUH 0.86 [0.51, 1.45]
SNUBH 0.77 [0.40, 1.48]
ASAN 0.83 [0.52, 1.34]
0.83 [0.61, 1.13]
Test*: Q= 4.59, df=2(p= 0.10), I²= 56.3%
0.99 [0.63, 1.55]
1.46 [0.95, 2.24]
0.75 [0.48, 1.16]
1.03 [0.70, 1.51]
Test*: Q= 0.27, df=2(p= 0.87), I²= 0.0%
1.05 [0.77, 1.43]
1.08 [0.76, 1.53]
1.17 [0.88, 1.56]
1.10 [0.92, 1.32]
Multivariate
Test*: Q= 0.28, df=2(p= 0.87), I²= 0.0%
SNUH 0.86 [0.51, 1.46]
SNUBH 0.69 [0.36, 1.32]
ASAN 0.79 [0.49, 1.26]
0.79 [0.58, 1.07]
Test*: Q= 4.93, df=2(p= 0.08), I²= 59.3%
1.00 [0.64, 1.57]
1.42 [0.92, 2.18]
0.71 [0.45, 1.10]
1.00 [0.67, 1.50]
Test*: Q= 0.39, df=2(p= 0.82), I²= 0.0%
1.04 [0.77, 1.41]
1.12 [0.78, 1.60]
1.19 [0.89, 1.59]
1.12 [0.93, 1.34]
0.25 0.5 1 4
0.25 0.5 1 4
0.25 0.5 1 4
